# P-2009. Initiative to Increase ASCVD Risk Assessment and Risk-associated Statin Utilization among PLWHIV in Infectious Diseases Clinic

**DOI:** 10.1093/ofid/ofaf695.2173

**Published:** 2026-01-11

**Authors:** Alex Belote, Jessica R Newman, Victoria Poplin, Megan Geraghty, Austin Price, Kelly Robertson, Megan Herrman, Lisa A Clough

**Affiliations:** University of Kansas Medical Center, Kansas City, Kansas; University of Kansas Medical Center, Kansas City, Kansas; University of Kansas Medical Center, Kansas City, Kansas; University of Kansas Medical Center, Kansas City, Kansas; University of Kansas Medical Center, Kansas City, Kansas; University of Kansas Medical Center, Kansas City, Kansas; University of Kansas Medical Center, Kansas City, Kansas; The University of Kansas Medical Center, Kansas City, Kansas

## Abstract

**Background:**

Atherosclerotic cardiovascular disease (ASCVD) is the leading cause of death in the U.S. In persons living with HIV (PLWHIV), the risk for development of ASCVD is twice that of the general population. Recent data from the REPRIEVE trial demonstrated that PLWHIV at intermediate ASCVD risk (5-20%) experienced decreased incidence of major adverse cardiovascular events when treated with moderate intensity statins. Recent guidelines for the use of antiretrovirals were updated to recommend moderate intensity statin in PLWHIV with an intermediate ASCVD risk. The aims of this quality improvement (QI) study were to increase identification of PLWHIV at an elevated risk for ASCVD who would benefit from statins, and to increase HIV provider recommendations/prescriptions for statin use in those at risk.

**Figure:**
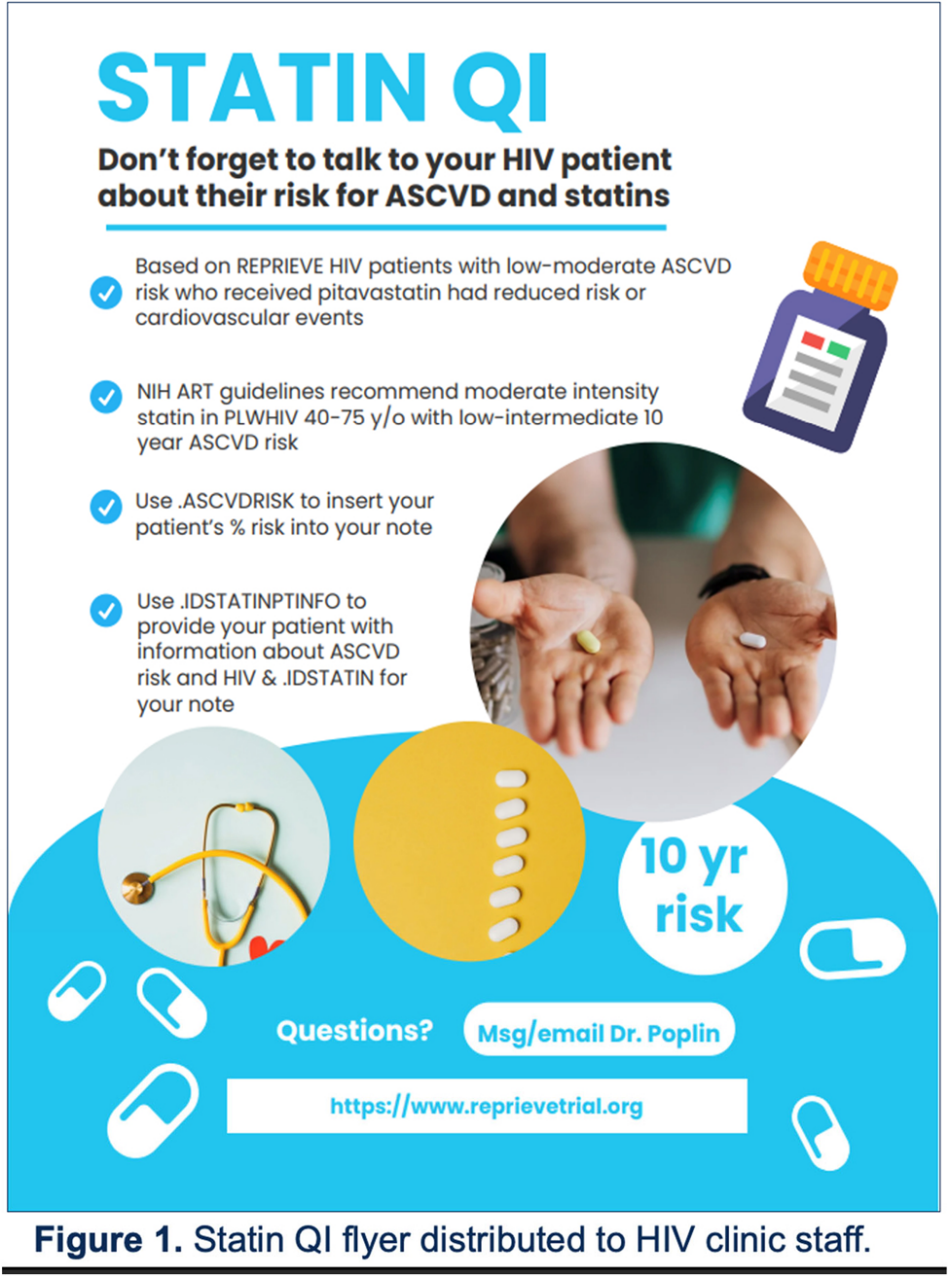

**Figure:**
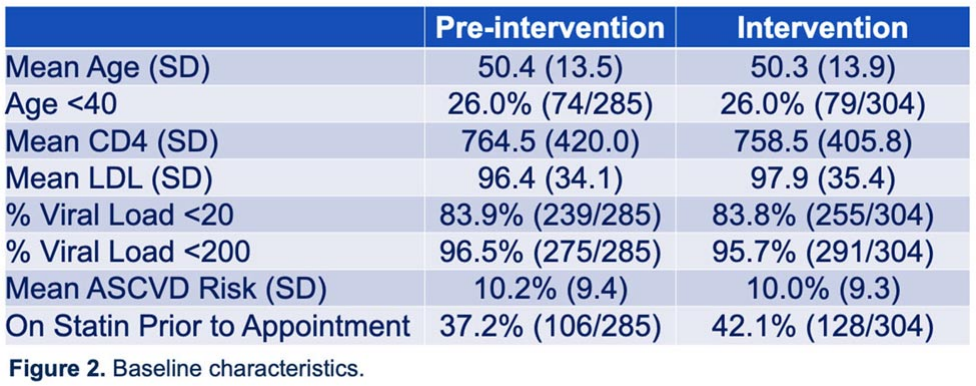

**Methods:**

We conducted a pharmacy-driven QI initiative in our HIV clinic. Over a 3-month intervention period in 2024, pharmacists calculated ASCVD risk, reviewed drug-drug interactions, and if appropriate, recommended statins to HIV providers. Information sheets were placed in the clinic (Figure 1). Project goals and interventions were presented to the ID department prior to initiation. Pre-intervention (Pre-I) data was obtained from 2023. Pre-I and intervention demographics, CD4-count, HIV viral load, LDL, and ASCVD risk scores were obtained.

**Figure:**
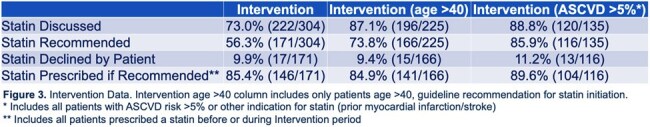

**Figure:**
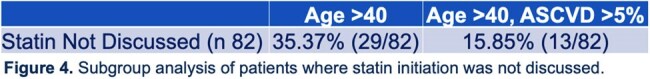

**Results:**

The pre-I and intervention groups had comparable age, CD4-count, HIV viral load, LDL, and ASCVD risk scores (Figure 2). In the intervention group, statins were discussed in 73% of appointments. When adjusting intervention data for age >40 (guideline age for statin initiation) or ASCVD risk >5%, statins were discussed 87.1% and 88.8% of the time, respectively. Statins were recommended more frequently in those age >40 or ASCVD risk >5%. Statins were prescribed if recommended for 85.40% of all appointments. The most cited reason for no prescription was “patient preference”.

**Conclusion:**

Our pharmacy-driven QI initiative increased identification of PLWHIV at an elevated risk for ASCVD, and statin recommendations/prescriptions. Statin prescriptions were increased to 85% in those recommended. This initiative provides data for ongoing use of ASCVD risk stratification and statin recommendation in HIV clinics to help combat ASCVD in PLWHIV.

**Disclosures:**

All Authors: No reported disclosures

